# Geocoding routinely collected administrative data to measure access to alcohol outlets in Wales

**DOI:** 10.23889/ijpds.v1i1.342

**Published:** 2017-04-19

**Authors:** Richard Fry, Sarah Rodgers, Scott Orford, Jennifer Morgan, David Fone

**Affiliations:** 1 Farr Institute, Swansea University; 2 Cardiff University

## Objectives

A substantial level of excess alcohol consumption results in a wide range of harm and the potential impact on health at the population level of a reduction in consumption is considerable. A proposed policy for reducing alcohol consumption is restricting the availability of alcohol through reducing the density of alcohol outlets. We set out to create a high spatial resolution alcohol outlet dataset suitable for evaluating longitudinal changes in chronic alcohol related conditions. 

## Approach

Requests were made for the names and location of all licensed alcohol outlets within each of the 22 Unitary Authorities in Wales, between Nov 2005 and Dec 2011. Data requested for each outlet consisted of: the date permission was granted or the licence became active, the licence expiry date or an indicated date of outlet closure, whether this premise is licensed for ON and/or OFF premise sales, the hours permissible to sell alcohol or general opening hours of the outlet and the type of premise as assigned by the LA if available. Our approach included collating, geocoding and manually matching alcohol outlet data received from each unitary authority for use in a longitudinal analysis of outlet density.

## Results

All authorities were able to provide an actual or approximate license issue date, allowing us to summarise the number of outlets annually. Several authorities were unable to provide precise outlet closure dates, so the date of the last interaction with the outlet was used to generate an approximate end date. One-half of the unitary authorities were able to provide the On/Off sales status of outlets, and 9 were able to provide opening hours. From these data we were able to geocode 53% (range 28% to 72% by local authority) using GIS, the remaining 47% were matched using Google products to verify and extract a precise geographic location.

## Conclusion

The collation and processing of retrospective alcohol outlet data was successfully completed to enable the building of a longitudinal exposure dataset. There was considerable variation between the unitary authorities in the quality of address data, and data related to the availability of alcohol, for example opening hours. The lack of address structure required us to devise a manual address matching process to capture the addresses that could not be geocoded. To aid future data linkage based evaluations to provide policy evidence in a timely manner, local government datasets should use standardised data fields, including addresses and Point-of-Capture address verification. 

